# Hippocampal Gene Expression in Zebra Finches Reveals Sex- and Subregion-Specific Topographies

**DOI:** 10.1523/ENEURO.0121-26.2026

**Published:** 2026-08-21

**Authors:** Alexander C. Taylor, Chinweike N. Asogwa, Changjiu Zhao, Farrah N. Madison

**Affiliations:** ^1^Department of Integrative Biology, University of Wisconsin-Madison, Madison, Wisconsin 53706; ^2^ Department of Molecular, Cell & Developmental Biology, University of California Santa Cruz, Santa Cruz, California 95064

**Keywords:** hippocampus, sex differences, songbird, transcriptomics

## Abstract

The hippocampus is a highly evolutionarily conserved brain region that supports spatial memory, stress responses, and social behavior across vertebrates. While mammalian hippocampal transcriptomics have been extensively characterized, comparative data from the avian hippocampus remain limited. Using RNA sequencing, we characterized hippocampal gene expression in zebra finches (*Taeniopygia guttata*) across sex, rostral–caudal subregions, and acute social conditions (Paired, Separated, Unpaired). Although acute social conditions produced only modest transcriptional effects, with 32 differentially expressed genes (DEGs) reaching significance, robust differential gene expression emerged across sex (2,148 DEGs) and rostral–caudal subregion (2,259 DEGs). Notably, 28% of sex-biased DEGs mapped to the Z chromosome, consistent with incomplete dosage compensation in the avian brain. Gene Ontology enrichment across sex highlighted SMAD signaling and transforming growth factor beta (TGF-β)/activin pathway regulation as repeated organizational features of the zebra finch hippocampal transcriptome. Region-specific DEGs converged on themes of membrane excitability and synaptic plasticity, while social condition effects were sex- and subregion-specific, with females demonstrating a more pronounced transcriptional response to pair bonding and mate separation than males, particularly in the caudal hippocampus. These findings establish a foundational molecular reference for the zebra finch hippocampus and position the songbird as a comparative model for investigating how sex, neuroanatomical subregion, and social context interact at the molecular level to shape hippocampal function across vertebrates.

## Significance Statement

The hippocampus plays a central role in memory, spatial navigation, and social behavior across vertebrates, yet comparative transcriptomic data from the avian hippocampus remain limited. Using RNA sequencing in zebra finches, we demonstrate that sex and rostral–caudal subregions are dominant predictors of hippocampal gene expression, outweighing the effects of social condition. Strong enrichment of Z-linked genes among sex-biased transcripts highlights incomplete dosage compensation as a key contributor to molecular sex differences in the avian brain. Social condition effects were sex- and subregion-specific, with females showing robust transcriptional responses to pair bonding and separation across subregions. These findings establish a foundational molecular reference for the songbird hippocampus and advance understanding of how sex- and region-specific gene expression shapes hippocampal function.

## Introduction

Animals living in groups must navigate complex social environments by distinguishing between familiar and unfamiliar conspecifics, identifying potential mates, and deciding when to engage in courtship or territorial defense. Coordinating these behaviors requires the ability to recognize and appropriately respond to social cues. Numerous brain regions have been investigated for their roles in regulating social behavior and promoting group cohesion and survival (Okuyama et al., 2014; Walum and Young, 2018). Advances in methodological approaches and an appreciation of the neural complexity underlying sociality have led to the identification of over thirty regions and subregions involved in the social behavior network (Goodson, 2005; Ko, 2017; Chen and Hong, 2018; Fernández et al., 2018; Wei et al., 2021; Wang et al., 2023; Phalip et al., 2024). Despite its role in cognition, context processing, and learning and memory processes that may play important roles in social decision-making, the hippocampus has only been incorporated into this network relatively recently (Rubin et al., 2014). Research has demonstrated that the hippocampus encodes socially relevant information such as a conspecific's spatial location (Omer et al., 2018), the distinction between familiar and unfamiliar individuals (Opendak et al., 2016), and social hierarchy relationships (Kumaran et al., 2012), suggesting that the hippocampus plays an important role in coordinating social interactions.

The mammalian hippocampus has been studied extensively, and much is known about the functional contributions of its subregions to behavior (Kesner et al., 2004; Levone et al., 2021; Alahmadi et al., 2023). In mammals, the hippocampus is functionally divided along a dorsal–ventral axis, with dorsal regions primarily contributing to cognitive and spatial processing, while ventral regions are more strongly associated with stress regulation and emotional processing (Fanselow and Dong, 2010). A similar functional division is present in the avian hippocampus, although it is described along a rostral–caudal axis [for review, see Smulders (2017); Li et al. (2024); Madison et al. (2024)]. Comparative studies suggest functional homology between these axes, with the rostral avian hippocampus contributing to spatial navigation and the caudal hippocampus mediating stress-related responses (Fanselow and Dong, 2010; Atoji et al., 2016; Bingman et al., 2017; Smulders, 2017). Although considerable progress has been made in characterizing the behavioral functions of the avian hippocampus, the genetic and transcriptomic mechanisms underlying sex- and region-specific differences remain poorly understood, presenting a significant gap given the value of avian models for comparative hippocampal research.

Despite limited knowledge of the transcriptomic mechanisms mediating avian hippocampal function, studies examining neuronal activation have demonstrated that hippocampal subregions respond differently to social stimuli. In zebra finches (*Taeniopygia guttata*), sex-specific differences in immediate early gene expression (a marker of neuronal activity) have been observed across several hippocampal subregions. Exposure to offspring odors resulted in higher c-Fos densities in the dorsolateral region, slightly lower densities in the dorsomedial region, and the lowest densities in the ventral hippocampus in males exposed to their offspring's odors (Golüke et al., 2019). Similarly, male zebra finches housed communally for 40 d exhibited increased [3H]-thymidine–labeled cell proliferation in the caudal hippocampus compared with singly housed birds and compared with the rostral hippocampus (Barnea et al., 2006). Together, these findings suggest that hippocampal subregions respond differently to changes in the social environment.

Additional work further supports sex-specific involvement of the hippocampus in social processing. Separation from a mate stimulates sexually dimorphic changes in corticosteroid receptor expression in the hippocampus. Male zebra finches demonstrated downregulation of both mineralocorticoid (MR) and glucocorticoid receptors (GR), while females upregulate MR expression (Madison et al., 2018). These findings suggest that males and females may differentially recruit hippocampal circuits when responding to disruptions in their social environment. Building on this work, the present study used RNA sequencing (RNA-seq) to examine sex- and region-specific differences in hippocampal gene expression in the zebra finch. Because social experience can influence neural gene expression, birds were housed under different social conditions (Paired, Separated, Unpaired) to assess whether social context modulates transcriptional profiles. By identifying transcriptional differences across hippocampal regions and between sexes, this study provides new insight into the molecular mechanisms that may contribute to variation in hippocampal function and social behavior.

## Materials and Methods

### Animals

Adult zebra finches were purchased from an avian breeder and housed in same-sex cages at the University of Wisconsin-Madison. Birds were housed in an indoor aviary in 49 × 95 × 51 cm cages on a photoperiod of 14L:10D (light/dark) and provided food and water *ad libitum*. After ∼8 weeks, birds were moved to mixed-sex cages with nest boxes to facilitate pair-bond formation (12 males and 12 females) and were monitored daily for pair-bonding behaviors. Stereotyped behaviors associated with pair bonding in this species, including clumping, allopreening, billing, and cohabitation, were recorded to verify pair bonds (Silcox and Evans, 1982; Remage-Healey et al., 2003; Madison et al., 2018). After 12 d of observation, confirmed paired and unpaired birds were transferred in groups of 2–3 pairs (4–6 birds per cage) to new cages of the same type in the same room to minimize stress associated with the housing change. Following a 72 h acclimation period, birds were assigned to one of three treatment groups: (1) **Separated**, females (*n* = 4; one female died after separation and was excluded from analysis) were removed from their bonded males (*n* = 5) and housed together in a single same-sex cage, while males were housed together in a single same-sex male cage; (2) **Paired**, bonded pairs (*n* = 5 males; *n* = 5 females) were briefly captures and restrained to match the handling disturbance experience by the Separated group and then returned to their home cages and remained housed with their partner (three pairs per cage, two cages); and (3) **Unpaired**, same-sex housed males (*n* = 2) and females (*n* = 2) that had not formed pair bonds were also briefly captured and restrained and then returned to their same-sex cages without change to their housing condition. After 24 h in their assigned social condition, brains were rapidly extracted, flash-frozen in powdered dry ice, and stored at −80°C. After 24 h in their assigned social condition, brains were rapidly extracted, flash-frozen in powdered dry ice, and stored at −80°C. This 24 h time point was selected based on prior research in zebra finches demonstrating that circulating corticosterone concentrations remain significantly elevated 24 h after pair separation but return to baseline upon reunion (Remage-Healey et al., 2003). Moreover, at 36 h postseparation, corticosterone concentrations remained elevated and significant changes in gene expression are detectable (Madison et al., 2018), suggesting that 24 h is sufficient time for meaningful transcriptional differences to be established following pair separation.

### Brain punches and RNA extractions

**Figure 1.**
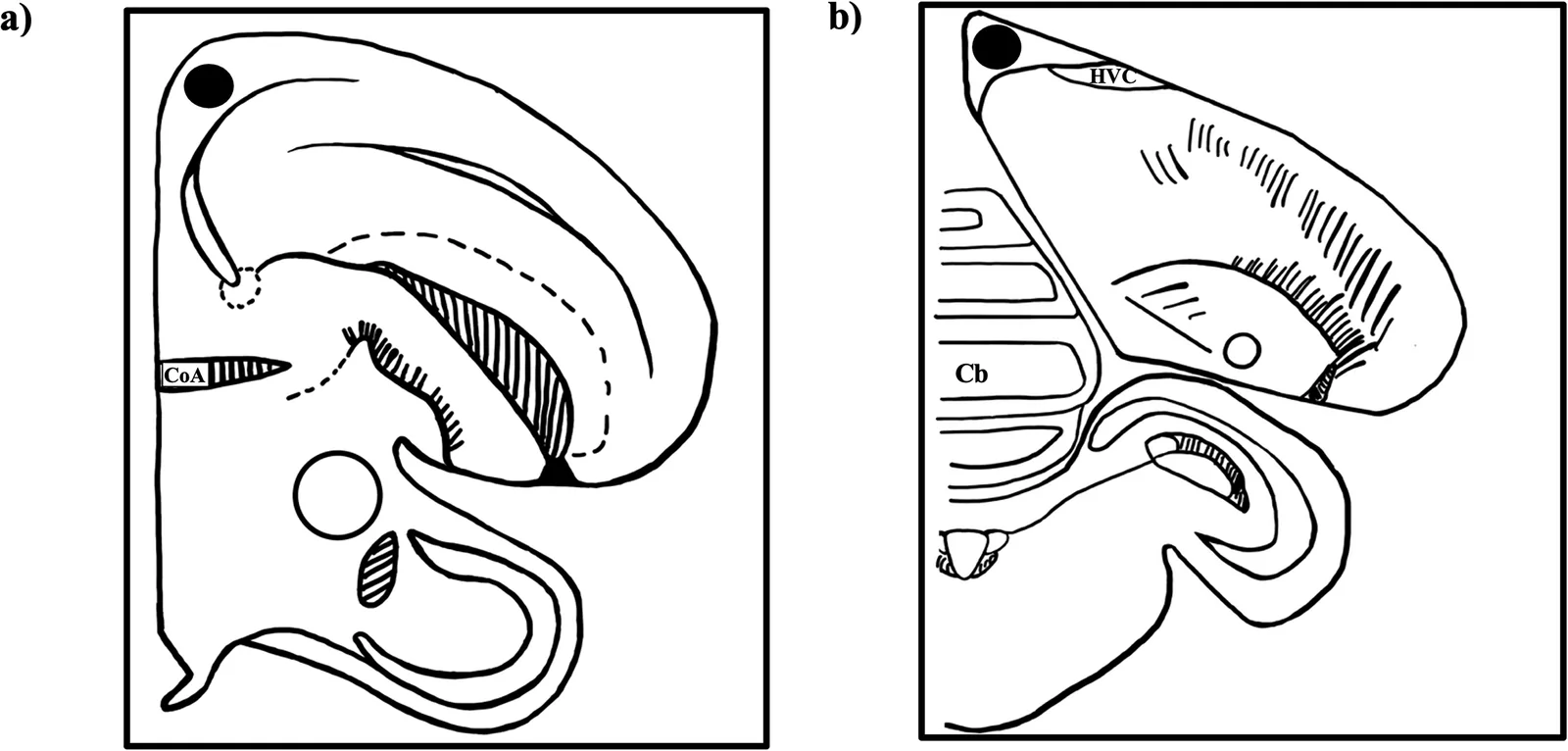
Schematic rendering of the zebra finch stereotaxic atlas plates illustrating the most rostral portion of the rostral (***a***) and caudal (***b***) hippocampal punches for RNA-seq. Black circles represent the area punched in each hemisphere for RNA-seq. Images adapted from Nixdorf-Bergweiler and Hans-Joachim Bischof, 2007. Anterior commissure (CoA); HVC (known as acronym); cerebellum (Cb).

Brains were sectioned at 100 µm thickness in the coronal plane using a cryostat (Leica, CM1950) referencing the zebra finch brain atlas for anatomical coordinates. Sections were mounted onto frozen glass slides in the cryostat set at −16°C. One-millimeter circular punches were collected from both hemispheres of each section using a Palkovits punch. Rostral hippocampal punches were obtained from sections spanning the anterior commissure to the first appearance of the cerebellum (Fig. 1*a*). Caudal hippocampal punches were collected from the first appearance of the cerebellum through sections where four distinct cerebellar lobes were visible (Fig. 1*b*). To minimize contamination from adjacent brain regions, hippocampal punches were taken from the dorsomedial region. This sampling strategy ensured that the same subregion was consistently represented across the rostral–caudal hippocampus and that only transcripts from this defined area were sequenced. Punches from the same individual and anatomical region were pooled and stored at −80°C for RNA-seq.

### RNA extraction and RNA-seq

RNA was extracted using the Aurum Total RNA Fatty and Fibrous Tissue Module (Bio-Rad Laboratories) following the manufacturer's protocol. Extracted RNA purity and concentration were quantified using a NanoDrop One Spectrophotometer and Agilent Tapestation RNA Screentape. Six of the 48 samples showed phenol contamination and were treated with the Monarch RNA Cleanup Kit (New England Biolabs, #T2030S) and passed RNA quality control. They were then stored at −80°C until submission to the University of Wisconsin-Madison Gene Expression Center Core for sequencing. Briefly, libraries were quantified utilizing an Agilent BioMek Synergy H1 Plate Reader and an assay on an Agilent Tapestation 4200 with a library range of ∼200–100 bp. The University of Wisconsin-Madison Gene Expression Center Core Facility (RRID:SCR_017757) prepared the RNA libraries, and the DNA Sequencing facility (RRID:SCR_017759) sequenced the libraries. Reads were trimmed using Skewer (Jiang et al., 2014) and mapped to the NCBI zebra finch RefSeq assembly bTaeGut1.4.pri (GCF_003957565.2) with Spliced Transcripts Alignment to a reference (Dobin et al., 2013). Read counts were quantified with RSEM (Li and Dewey, 2011).

### Principal component analysis

To visualize global patterns of transcriptional variation across samples, principal component analysis (PCA) was performed on log-counts per million (log-CPM) transformed expression data using the prcomp() function in R. PCA scores were plotted using the ggbiplot (Vu and Friendly, 2023) package, with samples colored by subregion (rostral, caudal) and shaped by sex to assess transcriptional clustering across groups.

### Differential gene expression analysis

Gene expression analyses were performed in R-Studio (v2024.12.1 + 563) with R (v4.4.1). Raw read counts and code for the following analyses are available at github.com/ATayl4908/ZB-DGE-analysis. Differential gene expression analysis was performed using edgeR (v. 4.2.0; Robinson et al., 2010). Genes with low expression were filtered out with the filterByExpr (Chen et al., 2016) function. Counts were normalized with the TMM method (Robinson and Oshlack, 2010). Samples were grouped by sex (male or female), treatment group (Paired, Separated, Unpaired), and brain region (rostral or caudal), resulting in 12 groups. Contrasts were constructed for biologically relevant comparisons, and genes were considered differentially expressed within a contrast if the FDR-adjusted *p* value is ≤0.05. Genes mapping to the W chromosome were retained in the analysis, as their absence in males produces notably large log-fold changes in male/female comparisons (Fig. 3*a*–*c*).

### Enrichment analysis

Enrichment analysis was performed with the clusterProfiler package in R (v4.4.1). Using the lists of differentially expressed genes (DEGs), Gene Ontology (GO) enrichment analysis was performed with the groupGO function (Yu et al., 2012), and overrepresentation analysis was performed with the enrichGO and enrichKEGG functions (Ashburner et al., 2000). Gene set enrichment analysis was also performed with the gseGO (Yu et al., 2012) function, which uses the entire list of present genes to determine enrichment. The DAVID Knowledge Base was used to further analyze GO and KEGG enrichments in selected comparisons (Huang et al., 2009; Sherman et al., 2022). For each contrast, the lists of all DEGs (up- and downregulated) were uploaded separately. The gene background was set to the list of all genes with sufficient read counts, as determined by the filterByExpr function in edgeR (Chen et al., 2016). Enriched GO terms within Biological Process, Cellular Component, and Molecular Function were retrieved. The GO_Fat run option was chosen rather than the default GO_Direct to improve specificity. Semantically similar enriched GO categories within each comparison were clustered and visualized using the GO-Figure! package (v1.0.2) in Python (v3.9.21; Miniforge v24.11.3-2).

## Results

### Overview of hippocampal transcriptomic variation by sex and subregion

**Figure 2.**
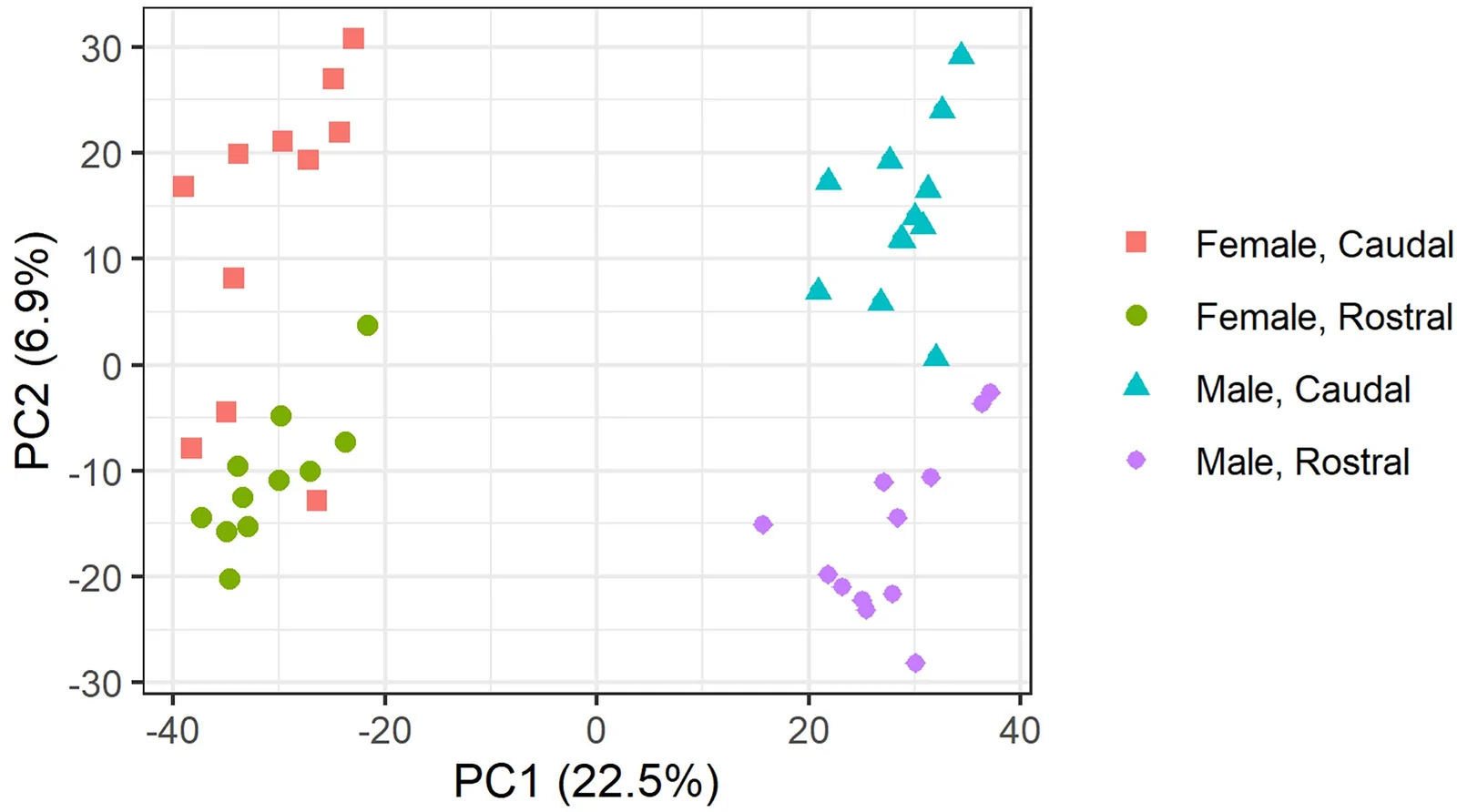
PCA of hippocampal gene expression by sex and subregion. PCA of hippocampal gene expression revealed separation of samples by both sex and subregion, with males demonstrating a more pronounced transcriptomic divergence between rostral and caudal hippocampus than females. PC1 and PC2 accounted for 22.5 and 6.9% of total variance, respectively.

PC1 explained 22.5% of variance and separated samples by sex, while PC2 explained 6.9% of variance and separated samples by rostral–caudal subregion within each sex, with all four groups: Female Caudal, Female Rostral, Male Caudal, and Male Rostral, forming distinct clusters. Notably, males demonstrated a more pronounced transcriptomic divergence between rostral and caudal hippocampus than females, consistent with the larger number of DEGs detected across the rostral–caudal axis in males than females (1,128 vs 478 DEGs; see below). These data suggest that sex is a dominant driver of transcriptomic variation in the zebra finch hippocampus, followed by rostral–caudal subregion (Fig. 2).

### Differential gene expression in the male and female hippocampus

**Figure 3.**
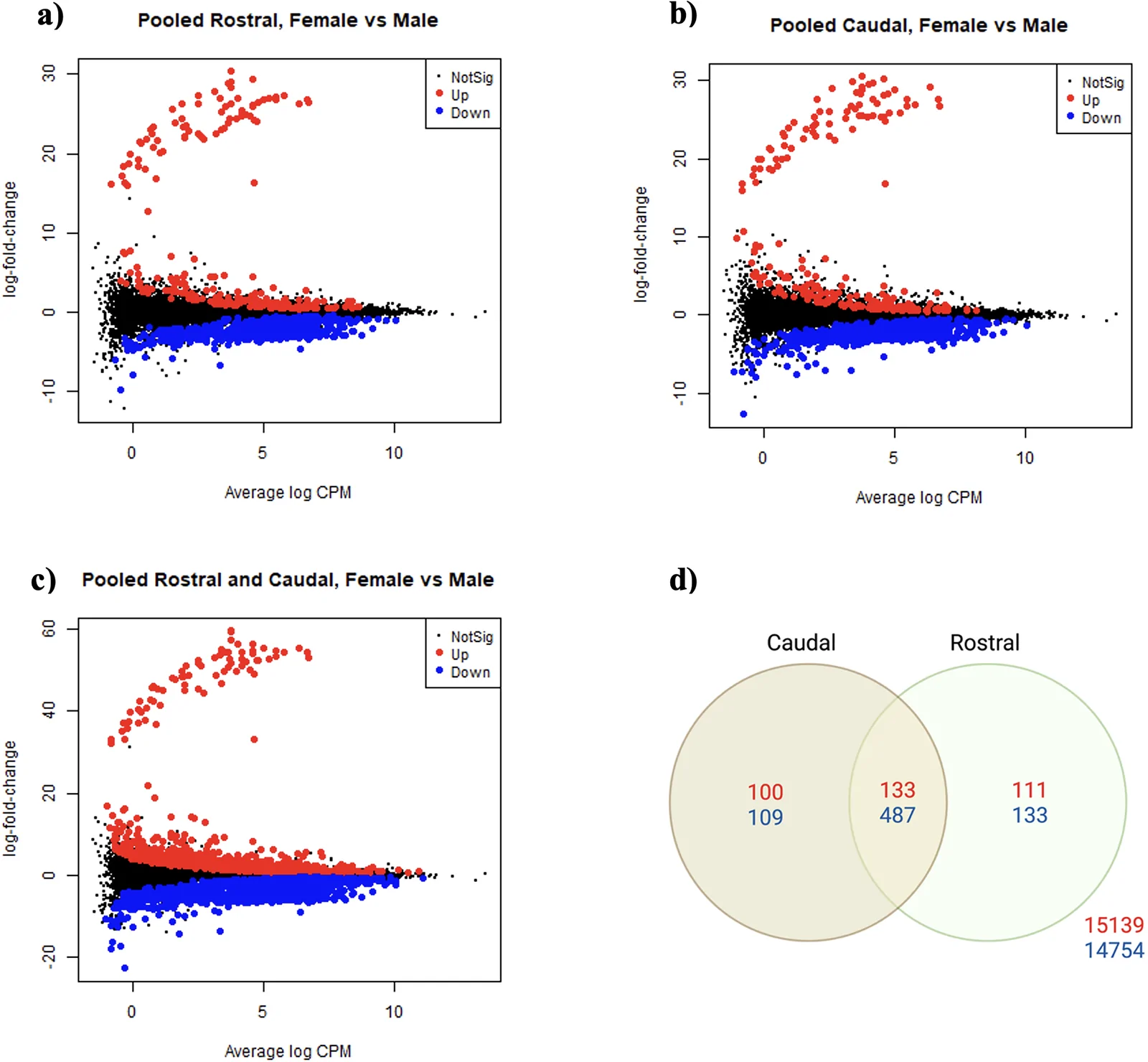
***a–c***, Differential gene expression between females and males in the (***a***) caudal, (***b***) rostral, and (***c***)pooled rostral–caudal hippocampus. Red dots represent genes with significantly higher expression in females; blue dots represent genes with significantly higher expression in males; black dots represent genes not reaching significance. The discrete cluster of red points with high log-fold changes reflects W chromosome-linked genes, which are absent in males and show notably large fold changes in female–male comparisons. ***d***, Venn diagram depicting overlap between sex-biased DEGs identified in the region-specific analysis of the caudal (left) and rostral (right) hippocampus (corresponding to ***a*** and ***b***, respectively). All genes shown are differentially expressed between females and males in at least one subregion. The red text indicates the number of genes with significantly higher expression in females; the blue text indicates higher expression in males. The intersection represents DEGs that are significant in both subregions. Note that the 2,148 DEGs identified in the fully pooled analysis (Fig. 3*c*) are not represented in Figure 3*d*, as that analysis sacrifices regional specificity in favor of statistical power. Significance threshold, FDR-adjusted *p* ≤ 0.05.

To maximize statistical power for detecting sex differences across the hippocampus, we first performed DGE analysis comparing males and females with rostral and caudal samples pooled, revealing 2,148 DEGs (Fig. 3*a*–*c*). Chromosomal annotation revealed that 601 of these DEGs (28%) were located on the Z chromosome, while only 79 (3.7%) were W-linked. Because only ∼5% of genes tested across the transcriptome are Z-linked, these results suggest a strong enrichment of Z-linked genes among the sex-biased DEGs. Notably, a subset of genes with large log-fold changes upregulated in females (visible as a distinct cluster in Fig. 3*a*–*c*) mapped to the W chromosome; as these genes are absent in the male genome, their elevated fold changes reflect chromosomal absence rather than differential transcriptional expression. To further characterize regional specificity of sex-biased expression, we performed region-specific comparisons of female versus male expression in the rostral and caudal hippocampus separately (Fig. 3*a*,*b*, respectively). Across these two analyses, 1,073 unique genes were differentially express between the sexes in at least one subregion. Of these, 209 DEGs were unique to the caudal hippocampus (100 upregulated in females, 109 upregulated in males), 244 were unique to the rostral hippocampus (111 upregulated in females, 133 upregulated in males), and 620 were shared across both subregions (133 upregulated in females, 487 upregulated in males; Fig. 3*d*). The additional ∼1,075 sex-biased DEGs identified in the pooled analysis but absent from the region-specific analyses likely represent genes whose expression differences are detectable with greater statistical power across the whole hippocampus but do not reach significance within either subregion individually. Among DEGs, 136 GO categories were enriched in females (relative to males), and 233 were enriched in males. Enriched GO categories include ATP biosynthetic process (GO:0006754), SMAD protein complex (GO:0071141), and cytokine receptor binding (GO:0005126; Fig. 4).

**Figure 4.**
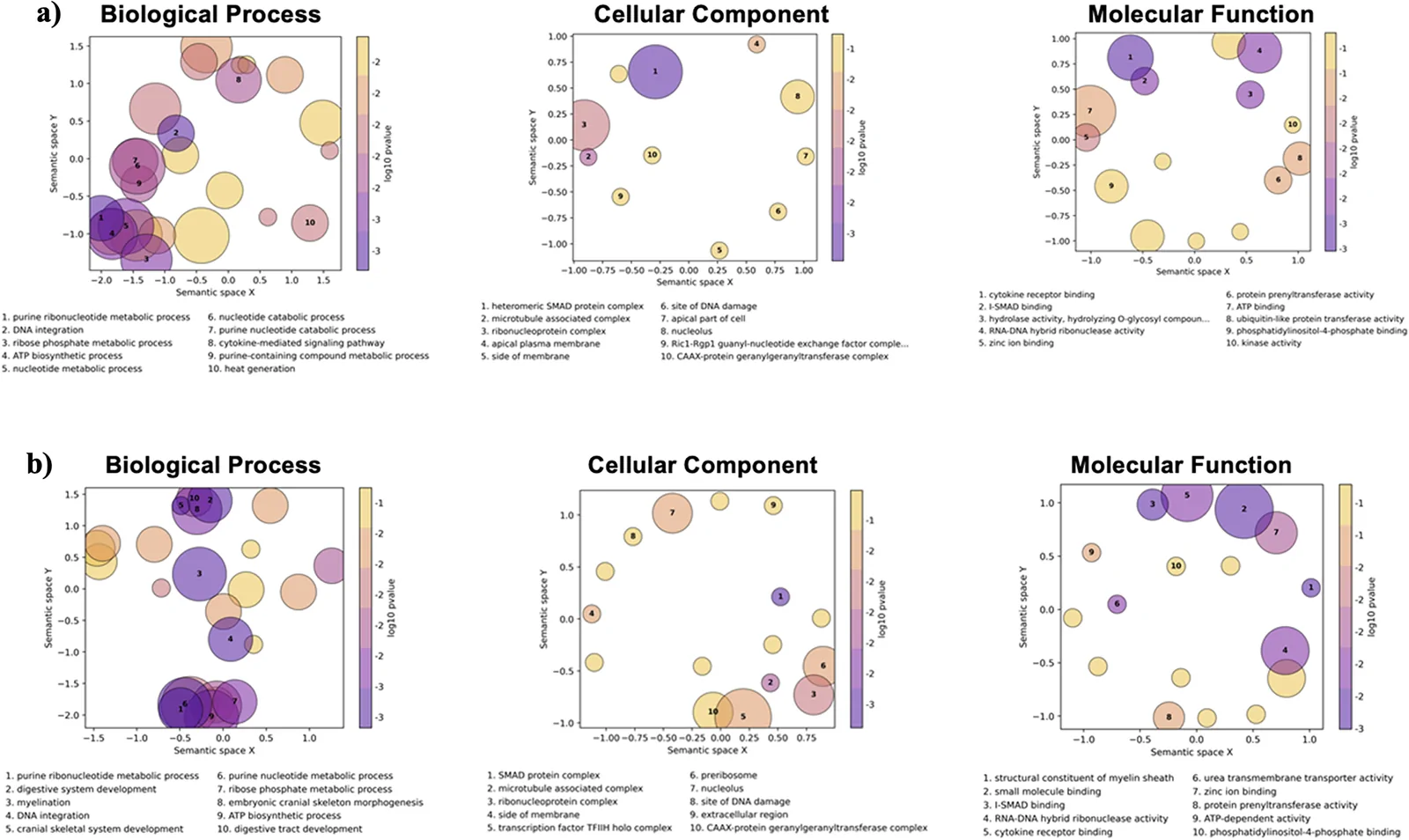
GO-Figure! plots of GO categories enriched among genes differentially expressed between males and females. ***a***, GO enrichment in sex differences within the rostral hippocampus. ***b***, GO enrichment within the caudal hippocampus.

### Differential gene expression in the rostral and caudal hippocampal extent

**Figure 5.**
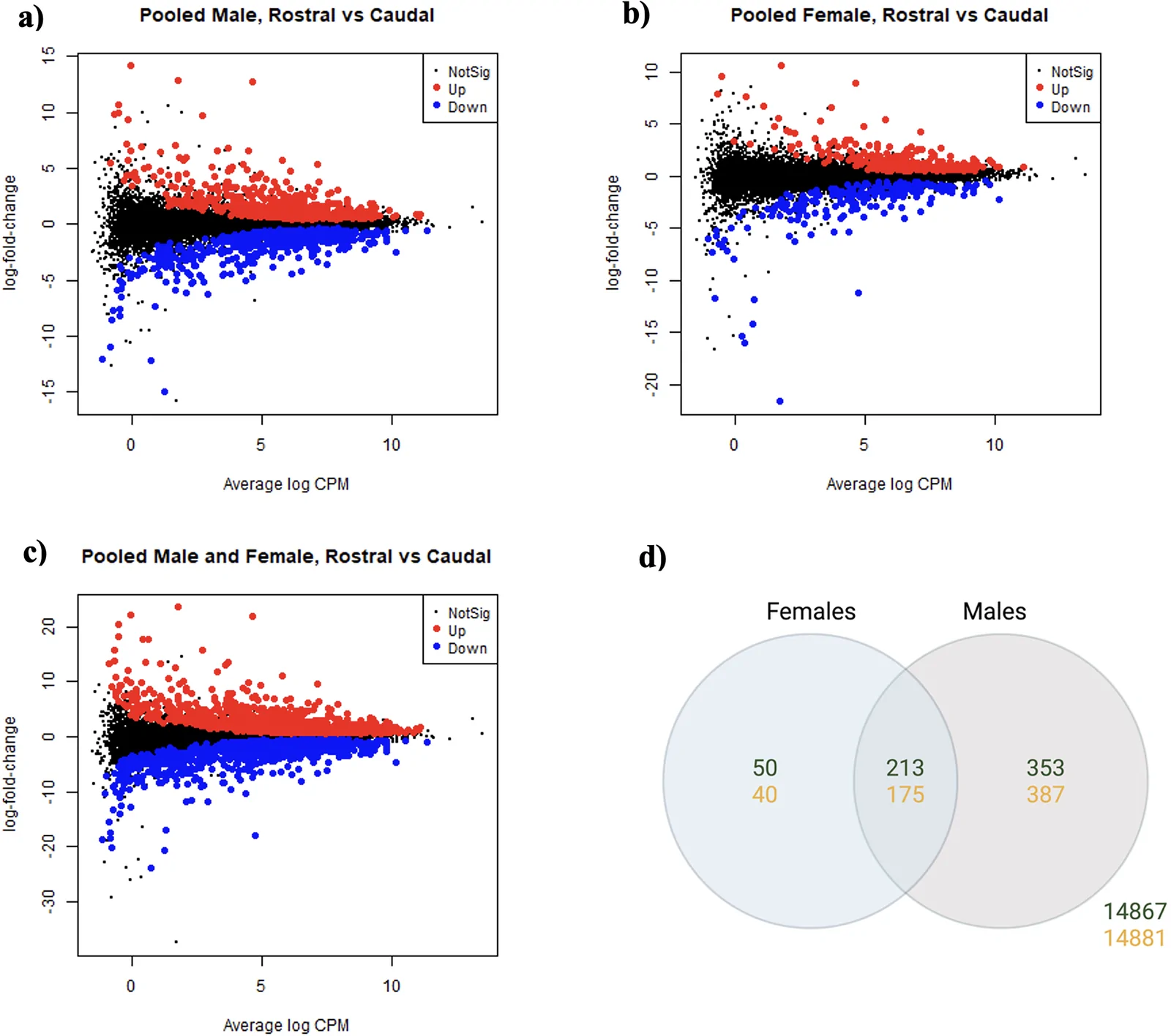
***a–c***, Differential expression between rostral and caudal hippocampus in (***a***) males and (***b***) females and (***c***) pooled males and females. Red dots represent genes with significantly higher expression in the rostral hippocampus; blue dots represent genes with significantly higher expression in the caudal hippocampus; black dots represent genes not reaching significance. ***d***, Venn diagram depicting overlap between rostral–caudal DEGs identified in the sex-specific analyses of females (left) and males (right; corresponding to ***b*** and ***a***, respectively). All genes shown are differentially expressed between the rostral and caudal hippocampus in at least one sex. Green text indicates the number of genes with significantly higher expression in the rostral hippocampus; yellow text indicates higher expression in the caudal hippocampus. The intersection represents DEGs reaching significance in both sexes. Note that the 2,259 DEGs identified in the fully pooled analysis **(*c***) are not represented in ***d***, as that analysis sacrifices sex specificity in favor of statistical power. Significance threshold, FDR-adjusted *p* ≤ 0.05.

To maximize statistical power for detecting rostral–caudal differences, we first performed DGE analysis comparing rostral and caudal samples with male and female samples pooled, revealing 2,259 DEGs (Fig. 5*c*). To further characterize sex-specific patterns of rostral–caudal expression, we performed sex-specific comparisons of rostral versus caudal expression in males and females separately (Fig. 5*a*,*b*, respectively). Across these two analyses, 791 unique genes were differentially expressed across the rostral–caudal axis in at least one sex. Of these, 90 DEGs were unique to females (50 upregulated in rostral, 40 upregulated in caudal), 740 were unique to males (353 upregulated in rostral, 387 upregulated in caudal), and 388 were shared across both sexes (213 upregulated in rostral, 175 upregulated in caudal; Fig. 5*d*). The additional ∼1,468 rostral–caudal DEGs identified in the pooled analysis but absent from the sex-specific analyses likely represent genes whose rostral–caudal differences are detectable with greater statistical power across the whole dataset but do not reach significance within either sex individually. Among genes differentially expressed across the rostral–caudal axis, 239 significantly enriched GO categories include neurogenesis (GO:0022008), activin receptor activity (GO:0017002), behavior (GO:0007610), and regulation of membrane potential (GO:0042391; Fig. 6).

**Figure 6.**
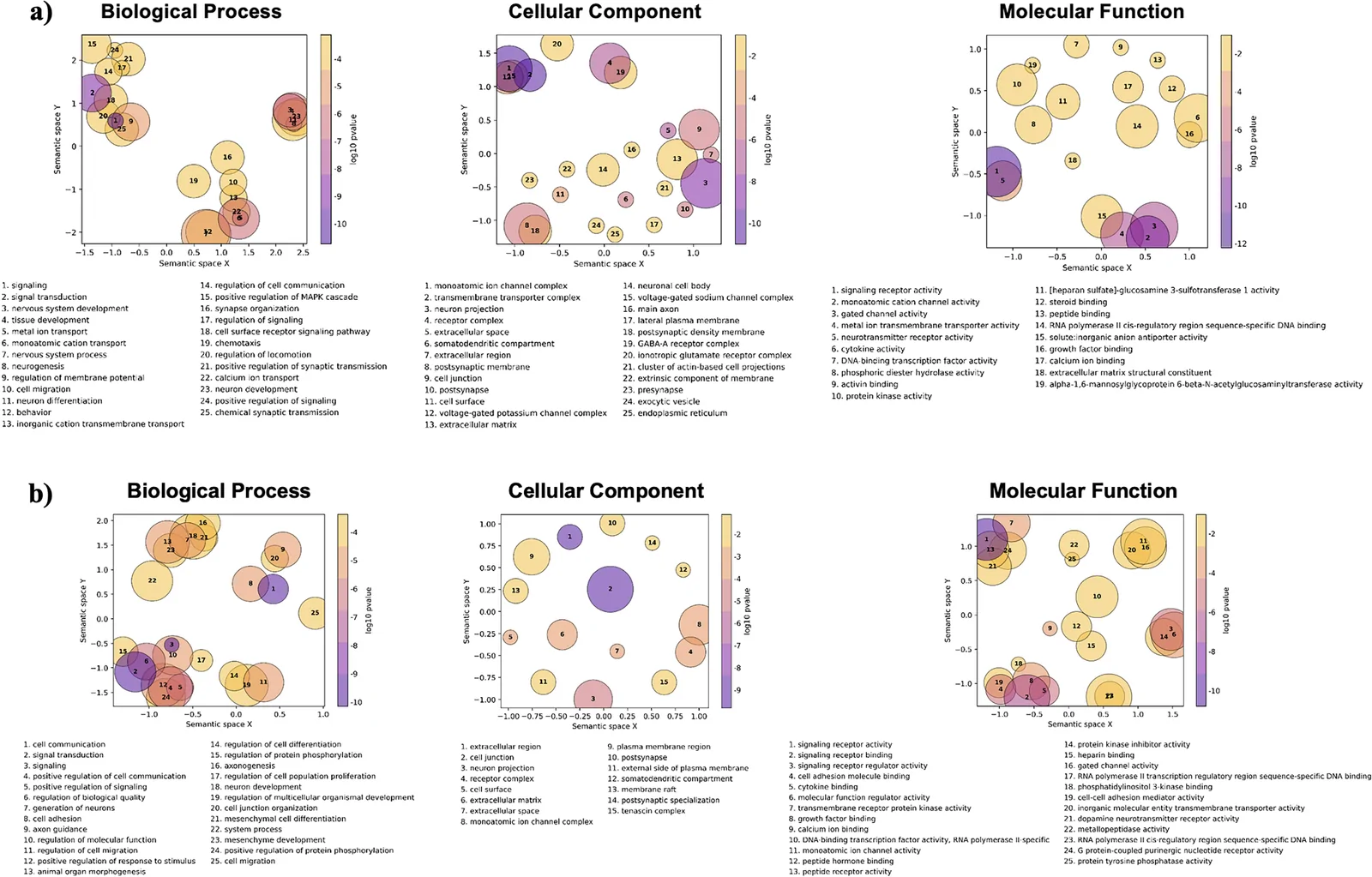
***a***, GO categories enriched in the rostral hippocampus (males and females pooled). ***b***, GO categories enriched in the caudal hippocampus (males and females pooled).

### Differential gene expression in Paired, Separated, or Unpaired male and female zebra finches

Thirty-two genes were differentially expressed across treatments (Paired, Separated, or Unpaired; Benjamini–Hochberg adjusted *p* ≤ 0.05). In Paired compared with Unpaired birds, four genes were downregulated in the male rostral and caudal hippocampus, while nine genes were downregulated in the female rostral and caudal hippocampus. In Separated compared with Unpaired males, five genes were downregulated in the male rostral and caudal hippocampus, while nine genes were downregulated in the female caudal hippocampus. Moreover, three genes were upregulated in the female caudal hippocampus. Finally, in Paired compared with Separated males, one gene was downregulated in the rostral hippocampus, whereas one gene was downregulated in the female caudal hippocampus. A Fisher's exact test comparing the number of DEGs between males and females across social conditions demonstrates that females have a significantly greater number of DEGs than males (Fisher's exact test, *p* = 0.0043). Of the 32 genes demonstrating significant differences in gene expression, 14 are uncharacterized (Table 1).

**Table 1.** DEGs by social housing

Comparison	Region	Male	Female
Paired versus Unpaired	Rostral	SPSB1 IGFN1	LOC121470831 ATP2A1 LOC115490949
	Caudal	SPSB1 IGFN1	LOC121470831 TET1 LOC115493804 MYMK LOC115493470 PTHLH
Separated versus Unpaired	Rostral	SPSB1 LOC121468908	None
	Caudal	SPSB1 IGFN1 LOC121468908	LOC115493804 C1H3orf52 LOC121470007 RIPK1 LOC121468025 MYMK LOC115493470 MZB1 LOC100223270 **NR1D1 NR1D2 PER3**
Paired versus Separated	Rostral	LOC115496831	None
	Caudal	None	LOC115495920

Genes in normal text were downregulated, while genes in bold text were upregulated.

## Discussion

Prior work demonstrated a sex-specific pattern of hippocampal gene expression in response to social disruption in zebra finches (Madison et al., 2018), suggesting that broader sex- and region-specific differences in baseline gene expression shape the hippocampus's response to environmental perturbation. This study used RNA-seq to characterize gene expression across sex, rostral–caudal subregion, and acute social condition (Paired, Separated, Unpaired) in zebra finches. Contrary to our expectations, acute changes in social condition revealed relatively modest transcriptional effects. However, when social condition was removed as a variable, robust sex- and region-specific DGE patterns emerged. These findings suggest that sex and hippocampal subregion explain more of the variance in the hippocampal transcriptome than acute changes in the social environment, providing a foundational molecular reference for the zebra finch hippocampus that complements mammalian hippocampal research. Furthermore, neither GR nor MR emerged among social condition DEGs, which could reflect true absence of regulation under these conditions, limited statistical power at the whole transcriptome level, or the inherent difference in sensitivity between candidate-gene approaches and unbiased transcriptome-wide analyses.

### Sex-specific differences in hippocampal gene expression

Although the hippocampus is among the most extensively studied brain regions in vertebrates, comparative transcriptomic data from the avian hippocampus remain limited. This gap is notable given that the avian hippocampus supports meaningful behaviors such as spatial navigation, food caching, and brood parasitism, offering a powerful framework for interpreting molecular findings (Clayton, 1998; Bingman et al., 2005; Guigueno and Sherry, 2017). Collapsing across social conditions, DGE analysis revealed 2,148 genes differentially expressed between males and females. Strikingly, 601 of these DEGs (28%) mapped to the Z chromosome, despite Z-linked genes comprising only ∼5% of the transcriptome-wide gene set tested, revealing a significant enrichment. Unlike mammals, in which X-inactivation buffers sex-chromosome dosage, birds exhibit only partial dosage compensation of Z-linked genes (Itoh et al., 2007; Mank and Ellegren, 2009). Thus, ZZ males are expected to express Z-linked genes at higher levels than ZW females in a largely gene-specific, hormone-dependent manner. Our data are consistent with this prediction and expand our understanding of incomplete dosage compensation, previously characterized in the brains of other species and in peripheral tissues (Uebbing et al., 2015), to the zebra finch hippocampus. Moreover, the W-linked DEG data are consistent with the limited genetic content of the passerine W chromosome (Smeds et al., 2015). These sex-specific patterns are consistent with recent transcriptomic work in neophobic and non-neophobic Eurasian tree sparrows, which similarly identified sex- and region-specific hippocampal gene expression, with the strongest effects in the rostral hippocampus of males and the caudal hippocampus of females (Lipshutz et al., 2026).

GO enrichment analysis emphasized the breadth of sex-specific transcriptional divergence: 233 GO categories were enriched in males compared with 136 in females. Across both rostral and caudal hippocampal regions, enriched Biological Processes involved DNA/RNA synthesis (purine ribonucleotide metabolic processes), while SMAD protein complex-related genes emerged as enriched in Molecular Functions (in both subdivisions). SMAD proteins are the canonical intracellular transducers of TGF-β/activin signaling, lending functional coherence to the top male-biased DEG: activin A receptor type 2A (ACVR2A). Activin A is a well-established neurotrophic and neuroprotective factor in the mammalian brain, and endogenous activin receptor signaling has been proposed to strengthen in the aging brain as a compensatory mechanism to attenuate cognitive decline (Nishiyama et al., 1997; Zheng et al., 2024). Interestingly, TGF-β signaling also regulates hippocampal neurogenesis in rodents (Wachs et al., 2006; Ware et al., 2026). Neurogenesis has also been described in the avian hippocampus (Hoshooley et al., 2005; Hoshooley and Sherry, 2007; Armstrong et al., 2020), suggesting that sex differences in ACVR2A expression may reflect differential rates of hippocampal structural plasticity between female and male zebra finches. ACVR2A was also differentially expressed in females, suggesting its regulation may be sex-specific in direction or magnitude rather than binary, a distinction warranting follow-up at the protein level. Additional male-biased DEGs included PTPRU, which regulates somatic and nuclear size in midbrain dopaminergic neurons (Liu et al., 2022) and may be involved in the maintenance and survival of midbrain dopaminergic neurons (Saucedo-Cardenas et al., 1998; Jacobs et al., 2009); DIRAS family GTPase 2 (DIRAS2), a small Ras GTPase highly expressed in the mammalian hippocampus that has been linked to ADHD susceptibility (Reif et al., 2011; Grünewald et al., 2018) and elevated expression in caudal serotonergic neurons in the developing mouse hindbrain (Grünewald et al., 2018); potassium voltage-gated channel subfamily Q member 4 (KCNQ4), a voltage-gated potassium channel expressed in the central auditory pathway in mammals (Kharkovets et al., 2000) whose male-biased expression in the zebra finch hippocampus may reflect sex-specific auditory hippocampal integration linked to song production; and SATB homeobox 1 (SATB1), a chromatin organizer that regulates neuronal plasticity dendritic spine morphology, synaptic plasticity, and immediate early gene expression in the mammalian hippocampus (Balamotis et al., 2012; Close et al., 2012; Turovsky et al., 2023). Top female-biased DEGs included discoidin CUB and LCCL domain containing 1 (DCBLD1), a transmembrane scaffolding protein involved in regulating neuronal apoptosis (Jia et al., 2025); interleukin 1 receptor subunit alpha 2 (IL13RA2), an interleukin receptor subunit implicated in regulating IL-1 signaling in the brain (Peters et al., 2013); and doublecortin-like kinase 1 (DCLK1), a doublecortin-like kinase involved in dendritic spine growth and microtubule bundling (Lipka et al., 2016; Murphy et al., 2023). Together, these top DEGs converge on themes of neuroplasticity, neuroimmune signaling, and circuit maintenance consistent with the notion that sex differences in hippocampal function in zebra finches are modulated via transcriptional mechanisms rather than acute hormonal fluctuations alone.

### Region-specific differences in hippocampal gene expression

While debate continues regarding the precise number of subdivisions in the avian hippocampus, functional and anatomical evidence support the homology between the rostral/dorsal and caudal/ventral regions (Bingman, 1992; Colombo et al., 2001; Atoji et al., 2016). Comparing rostral and caudal hippocampus across pooled males and females revealed 2,259 DEGs, confirming robust molecular differentiation along the rostral–caudal axis. Comparing the two regions within males revealed 1,128 DEGs, while the same comparison in females yielded 478 DEGs, suggesting that rostral–caudal transcriptional variance is substantially greater in males, consistent with the male-biased rostral hippocampal pattern reported in Eurasian tree sparrows (Lipshutz et al., 2026). GO analysis for rostral versus caudal comparisons highlighted 239 significantly enriched categories, including neurogenesis (GO:0022008), activin receptor activity (GO:0017002), behavior (GO:0007610), and regulation of membrane potential (GO:0042391). These categories are consistent with functional specialization in membrane excitability and synaptic communication along the hippocampal rostral–caudal axis.

The top region-specific DEGs (pooled males and females) included ACVR2A and DCBLD1, genes also prominent in the sex-specific analysis, as well as distinct subgroup of the Ras family member 2 (DIRAS2) and potassium voltage-gated channel subfamily Q member 4 (KCNK12). The recurrence of ACVR2A and DIRAS2 across both sex and regional comparisons is noteworthy, suggesting that these genes may serve as key organizational nodes in the zebra finch hippocampal transcriptome, with their expression varying along multiple biological axes simultaneously. KCNK12 encodes THIK-2, a two-pore domain potassium channel that regulates neuronal excitability and resting membrane potential in vitro (Renigunta et al., 2014). The top five DEGs shared across all rostral–caudal comparisons, regardless of sex, were Spindlin-Z, transcriptional endoplasmic reticulum ATPase (TERA/VCP/p97), activated RNA polymerase II transcriptional coactivator p15, microtubule-associated protein 1B (MAP1B), and heterogeneous nuclear ribonucleoprotein K (hnRNP K). TERA/VCP/p97 is implicated in multiple neurodegenerative disorders, including frontotemporal dementia and familial amyotrophic lateral sclerosis (Tang and Xia, 2016), while MAP1B plays a role in axon growth and in anchoring GABA_c_ receptors to the cytoskeleton (Takei et al., 1997; Billups et al., 2000). Of particular functional interest is hnRNP K, an RNA-binding protein that regulates dendritic spine density and long-term potentiation in mammalian hippocampal neurons (Folci et al., 2014). Moreover, it is mainly expressed in the ventral hippocampal neurons, with decreased expression resulting in stress-induced depression-like behaviors (Zhuang et al., 2023). Its rostral–caudal differential expression in the zebra finch hippocampus suggests that region-specific modulation of synaptic plasticity may be a conserved mechanism across vertebrates. These region-specific transcriptional patterns provide molecular support for the functional rostral–caudal specialization of the avian hippocampus and open new avenues for investigating how subregional gene expression contributes to distinct hippocampal circuit dynamics.

### Social condition influences hippocampal gene expression in a sex- and region-specific manner

In contrast to the large-scale transcriptional differences observed across sex and subregion, differential gene expression across social conditions (Paired, Separated, Unpaired) was comparatively limited, with only 32 genes reaching significance. This modest transcriptional response to acute social rearrangement is consistent with findings in other systems, suggesting that short-term environmental perturbations may be insufficient to drive broad transcriptomic changes (Stankiewicz et al., 2015; Uren Webster et al., 2018). Nonetheless, the pattern of responses was informative. More genes were differentially expressed in females than males across social conditions, and female responses were preferentially localized to the caudal hippocampus. This is consistent with previous evidence of sex-specific hippocampus sensitivity to stress, including evidence of reduced hippocampal proliferation in males relative to females in rodents, and with the proposed role of the caudal/ventral hippocampus in stress-related processing (Hillerer et al., 2013; Brydges et al., 2018; Kwon et al., 2025; Grace and Uliana, 2026).

When comparing Paired to Unpaired males, SplA/ryanodine receptor domain and SOCS box containing 1 (SPSB1) and immunoglobulin-like and fibronectin type III domain containing 1 (IGFN1) were significantly downregulated in both rostral and caudal hippocampus. SPSB1 negatively regulates transforming growth factor beta (TGF-β) signaling (Liu et al., 2015), a pathway that governs cell proliferation, differentiation, and survival (Massagué, 1998). Its downregulation in paired males may therefore disinhibit TGF-β activity, potentially promoting hippocampal cell survival or plasticity that helps maintain a stable pair bond. SPSB1 has also been reported to be upregulated following ethanol exposure in the mouse brain (Treadwell and Singh, 2004; Kleiber and Singh, 2009), suggesting a broader role in stress-responsive cellular regulation. IGFN1 is expressed in the hippocampus, but its neural function remains poorly characterized. It is underexpressed in glioblastoma (Lu et al., 2022), suggesting possible roles in cell survival pathways. In Paired compared with Unpaired females, downregulation of ATP2A1 was observed in the rostral hippocampus. Although ATP2A1 is primarily characterized in the context of skeletal muscle function (Pan et al., 2003), its expression in the hippocampus warrants further investigation. In the female caudal hippocampus, TET1, a dioxygenase that promotes DNA demethylation and is required for hippocampal neurogenesis (Guo et al., 2011; Zhang et al., 2013; Shuang et al., 2024) was significantly downregulated. TET1 inhibition enhances memory consolidation and storage (Kumar et al., 2015), while its overexpression in the hippocampus promotes anxiety-like behavior and fear memory (Kwon et al., 2018), suggesting that its downregulation in paired females may reflect memory encoding associated with the pair bond. Myomaker, myoblast fusion factor (MYMK) and parathyroid hormone-like hormone (PTHLH) were also downregulated in the female caudal hippocampus, while MYMK is considered largely muscle-specific, PTHLH encodes PTHrP, which has neuroprotective effects against excitotoxicity (Chatterjee et al., 2002) and modulates neuroinflammatory responses following traumatic brain injury (Funk et al., 2001). Together, these findings reveal sex-specific transcriptional changes in the hippocampus associated with pair bonding, with genes involved in cellular signaling, epigenetic regulation, and neuroprotection emerging as potential molecular substrates underlying the formation or maintenance of the pair bond.

When comparing Separated to Unpaired birds, males again showed downregulation of SPSB1 (rostral) and SPSB1/IGFN1 (caudal), the same as in the Paired versus Unpaired comparison, suggesting that any disruption of the baseline unpaired state, whether through pairing or separation, engages similar hippocampal molecular responses in males. Separated females showed no rostral hippocampal DEGs but exhibited downregulation of receptor-interacting serine/threonine kinase 1 (RIPK1), marginal zone B and B1 cell-specific Protein (MZB1), MYMK, and chromosome 1 C3orf52 homolog (C1H3orf52) in the caudal hippocampus, while period3 (PER3), nuclear receptor subfamily 1 group D member 1 (NR1D1), and nuclear receptor subfamily 1 group D member 2 (NR1D2) were upregulated. RIPK1 mediates inflammatory and cell death signaling (Degterev et al., 2008; Christofferson et al., 2012; Najjar et al., 2016), and its expression is elevated in Parkinson's disease models (Qiao et al., 2023); its downregulation in separated females may reflect dampened neuroimmune reactivity in the absence of a partner. The upregulation of PER3 is consistent with its reported induction following alcohol and restraint stress in rodents (Wang et al., 2012). NR1D1 and NR1D2, nuclear receptor transcription factors that modulate circadian rhythmicity, were also upregulated in separated females. NR1D1 upregulation in the female dorsal hippocampus has been reported following maternal separation in mice, where it was associated with enhanced social behavior and resilience to early-life stress (Ryabushkina et al., 2020). Furthermore, NR1D1 and NR1D2 have been shown to reduce brain injury and improve neuronal survival following traumatic brain injury, effects that are reversed by pharmacological inhibition (Zheng et al., 2022; Darmanto et al., 2024). The upregulation of these circadian and stress-responsive transcription factors in the female caudal hippocampus following mate separation suggests that females mount a more robust neuroendocrine and circadian transcriptional response to social disruption than males, consistent with the significantly greater number of DEGs observed in females across social conditions (4 vs 18 DEGs; Fisher's exact test, *p* = 0.0043).

### Conclusions

Together, these data provide a comprehensive transcriptomic characterization of the zebra finch hippocampus across sex, rostral–caudal subregion, and acute social condition. The predominance of sex- and region-specific effects over social condition effects highlights that natural baseline mechanisms exert greater influence on the hippocampal transcriptome than do short-term environmental perturbations. The strong enrichment of Z-linked genes among sex-biased DEGs highlights the contribution of incomplete dosage compensation to molecular sex differences in the avian brain, independent of gonadal hormones. Convergent patterns across sex and region comparisons, particularly involving ACVR2A, DIRAS2, and SMAD signaling, point to TGF-β/activin pathway regulation as a recurrent organizational feature of the zebra finch hippocampal transcriptome. Future research employing single-cell transcriptomics, targeted gene manipulation, and behavioral paradigms will be essential to determine whether these transcriptional differences reflect variation in cell-phenotype, cell-intrinsic gene regulation, or both and to link them to social behaviors, including mate-pair formation and maintenance. This study positions the songbird hippocampus as a tractable comparative model for investigating how sex, neuroanatomical subregion, and social context interact at the molecular level to shape hippocampal function across vertebrates.

## Synthesis

Reviewing Editor: Luke Remage-Healey, University of Massachusetts Amherst Decisions are customarily a result of the Reviewing Editor and the peer reviewers coming together and discussing their recommendations until a consensus is reached. When revisions are invited, a fact-based synthesis statement explaining their decision and outlining what is needed to prepare a revision will be listed below. The following reviewer(s) agreed to reveal their identity: Colin Saldanha.

The reviewers are seeking more clarity in the description of the experimental design, including social housing treatment conditions, brain micropunching areas, timing of housing conditions and whether it can really be inferred as a social difference, and formal statistical reckoning of the differentially-expressed genes.

Specifically related to the last point: Some further statistical analysis is warranted before concluding that there are sex differences in gene expression based on social housing condition. The reported merging of DEGs is not common practice and the authors should report the DEGs of each statistical comparison separately.

Reviewer comments and critiques are below:

REV 1:

The current manuscript describes the results of an RNAseq study that examined changes in gene expression in the hippocampus (HP) of zebra finches. The avian HP is not as well studied as its mammalian counterpart, but what we know strongly suggests homology of neuroanatomical and functional characteristics between the HP of both classes of vertebrate. This study used punches of HP tissue from the rostral vs caudal HP in male or female zebra finches that were part of three socially distinct groups. The latter were Paired, Separated, or Unpaired. The data suggest a modest number of differentially expressed genes (DEG) due to social housing, but when this constraint was removed, strongly DEG appeared across sex and rostro-caudal extent of the HP. The experimenters then use GO analyses and chromosome mapping to determine the kinds of gene pathways DEG, and the location of these genes on the chromosome. The data suggest that sex-driven DEG strongly map onto the Z chromosome relative to the W. Across it's rostro-caudal extent over a 1000 DEG were discovered in males compared to less than half that amount in females.

I have a few comments and questions.

1. In their description of the location of punches, Figure 1 depicts a single punch from each HP level per section. Is that correct? Also, the figure shows the punches coming from the dorsomedial component of the HP. Is that correct? If these statements are correct, the authors are urged to edit the methods so that the reader knows that this pattern of punches results in the same sub-area of the HP represented across its rostrocaudal extent, and that only transcripts from this area were sequenced. This would add specificity to the description and clarify the strength of the approach to the reader.

2. In a similar vein, I realize that there was normalization conducted later, but it would help the reader to know how much tissue was extracted across treatment groups.

3. I am a bit confused with the description of the social housing groups. Were the separated females housed in the same sex unpaired females or separately in a same-sex cage of only separated females? Regardless, is it the case that the separated males were housed in their paired cages (separate from each other) and the separated females housed in groups? Please clarify since if different, this may be an issue that needs to be considered in the statistical comparisons.

4. The described results for the social housing groups are unclear to me. Perhaps editing the prose may help? In line 179, instead of ,"In paired compared to unpaired males,.......while 9 genes were downregulated in the female rostral and caudal HP."

Is what the authors mean, "In paired compared to unpaired birds, 4 genes were downregulated in the rostral and caudal HP, and 9 genes were downregulated along the rostrocaudal HP in females." I think the latter is easier to understand(?). Similar for the next two comparisons.

REV 2:

I reviewed "Hippocampal gene expression in zebra finches reveals sex- and subregion-specific topographies" (eN-NWR-0121-26), which uses bulk RNAseq to summarize patterns of differentially expressed genes between the caudal and rostral hippocampus, in females and males, across three different housing conditions - paired, separated, and unpaired. The finding that many genes are differentially expressed between the rostral and caudal hippocampus supports a recent hypothesis for functional specialization of these brain regions, which may be analogous to the dorsal-ventral axis in mammals (e.g. Madison et al, 2024). I think the manuscript could benefit from additional visualization of results, and I have some suggestions. The finding that many genes are differentially expressed between sexes is expected in birds, due to incomplete dosage compensation on the Z chromosome as well as W-limited genes in females. I would caution the authors to take care in concluding which sex-specific results are biologically (and statistically) meaningful to the study's social design, versus due to genetic architecture.

L29-30: From Table 1 I see that females have qualitatively more DEGs under these different housing conditions - but is this significantly different between sexes? A Fisher's exact test would be helpful for quantification here. It's important not to overstate a sex difference if it isn't statistical. And what is meant by 'coordinated transcriptional responses' here? What does coordination mean in this context? (Same question applies to L355).

L102-105: Please provide sample sizes for these three treatment groups, for each sex. It is probably around n = 4 for group? That is rather small for differential expression analysis, but I understand that these kinds of studies are challenging.

L106: What is the justification for only 24 hours in these treatment conditions? That seems like not long enough time. The DEGs could result from stress in changing housing, rather than to the specific type of housing treatment.

L141: Some more information would be useful about what cutoffs were used to determine whether genes were differentially expressed, i.e. a particular log2foldchange, or the alpha level of the p-value, or both?

As another form of visualization, it could be useful to see a PCA of gene expression for brain regions and sex categories. For instance, this could show whether more variance in the data is explained by brain region or sex.

L159-160: Why were the rostral and caudal DEGs pooled between regions and between sexes in this results statement? I do not find that combination informative. It would be useful to know about the patterns for each brain region and for each sex in the results. From Figure 2d, this seems more like ~200 DEGs? A barplot could be useful to visualize the number of differentially expressed genes between brain regions and sexes.

Another possible visualization could be to show sex differences across brain regions, and to specifically visualize Z-linked and W-linked genes.

Perhaps I am misunderstanding Figure 2d though - it would be useful to know from the caption what the intersection of the Venn Diagram represents - genes with similar expression patterns in the rostral and caudal hippocampus? A similar concern applies to Figure 3d - what does the intersection of the Venn Diagram show? I am more used to Venn Diagrams depicting expressed genes, not DEGs, such that the overlap represents genes that are NOT differentially, expressed, and the separate part of the circle depicts genes that ARE differentially expressed. See Figure S3 from Lipshutz et al. 2026 Mol Ecol

Figure 2a-c: What is the cluster of red genes with very high log2fold change? The figure caption says that these genes are ones with higher expression in females - are these the 79 W-linked genes? It would make sense, since they are not found in males.

L190: Following Madison et al. 2018, were GR and MR in the lists of DEGs? It could be interesting to think about candidate vs. whole-transcriptome studies.

L202: I recommend reading Lipshutz et al. 2026 Mol Ecol - this study found sex and region specific differences in hippocampal gene expression in Eurasian tree sparrows.

L296-299: Please provide citations for this statement, since it references findings from other systems.

L301-304: As above, please provide citations for this statement, since it references findings from other systems.

Please check the reference formatting. For instance, Madison et al. 2018 is missing a date and some words are merged together. Madison et al. 2024 is completely missing from the references.
